# Comparison of pregnancy outcomes in uterine fibroids following high intensity focused ultrasound ablation vs. laparoscopic myomectomy: a propensity score-matched observational study

**DOI:** 10.3389/fsurg.2026.1767535

**Published:** 2026-03-02

**Authors:** Nenghuan Tang, Jie Yu, Wei Ran, Chunling Fang, Can Luo, Li Hu, Fan Xu, Yuhua Zeng

**Affiliations:** 1 Department of Obstetrics and Gynecology, Affiliated Hospital of North Sichuan Medical College, Nanchong, Sichuan, China; 2 Department of Clinical Medical College, North Sichuan Medical College, Nanchong, Sichuan, China; 3 Department of Obstetrics and Gynecology, The Affiliated Nanchong Central Hospital of North Sichuan Medical College, Nanchong, Sichuan, China; 4 Department of Gynecology, Shanghai First Maternity and Infant Hospital, School of Medicine, Tongji University, Shanghai, China

**Keywords:** high-Intensity focused ultrasound ablation, laparoscopic myomectomy, pregnancy outcomes, risk, uterine fibroids

## Abstract

**Purpose:**

To compare the pregnancy outcomes of patients with uterine fibroids (UFs) who underwent high-intensity focused ultrasound ablation (HIFU) vs. laparoscopic myomectomy (LM).

**Materials and methods:**

Patients with UFs and fertility undergoing HIFU or LM from January 2020 to June 2023 were included into this retrospective study. The primary outcomes were pregnancy outcomes including the natural pregnancy rate and the median time to pregnancy. Propensity score matching (PSM) was implemented to evaluate the primary outcomes. The secondary outcomes were the risk factors for natural pregnancy among patients with UFs receiving HIFU or LM.

**Results:**

In total, 171 patients were recruited in the HIFU group, while 122 in the LM group. Of them, 60 cases (60/293; 20.5%) achieved pregnancy, including 43 receiving HIFU whereas 17 undergoing LM. After PSM, 75 pairs were acquired. Difference in natural pregnancy rates was not statistically significant between the two groups (HIFU vs. LM: 13.3% vs. 14.7%; *p* = 0.841). Meanwhile, the interval period of pregnancy was of significant difference between the two groups [HIFU vs. LM: 13.0 (9.5–14.75) months vs. 17.0 (15.0–25.0) months, *p* = 0.002]. Moreover, as revealed by multivariate logistics regression analysis, age [odds ratio (OR) = 0.899, 95% confidence interval (CI): 0.835–0.969, *p* = 0.005] and classification of the main fibroids (intramural or submucous vs. subserous: OR = 0.379, 95%CI: 0.158–0.910, *p* = 0.030) were the independent factors affecting the natural pregnancy among women with UFs who underwent HIFU or LM.

**Conclusions:**

HIFU demonstrated comparable postoperative pregnancy rates to LM and was associated with a shorter median time to pregnancy, suggesting that HIFU may be a potential fertility-sparing treatment for women with UFs. Additionally, age and the classification of main fibroids were identified as the independent factors influencing postoperative pregnancy rates in patients undergoing treatment.

## 1 Introduction

Uterine fibroids (UFs) are the most commonly seen benign disease in women of childbearing age, which may cause numerous symptoms, such as abnormal uterine bleeding and pelvic discomfort, potentially leading to fertility issues (1). Recent studies have indicated that 2%–3% of UFs may be the considerable factor affecting fertility (2). Myomectomy and high-intensity focused ultrasound ablation (HIFU), as the prevalent fertility-preserving treatment options, have been extensively applied in clinical practice (3). However, data concerning their effects on pregnancy outcomes are scarce.

Myomectomy has been widely demonstrated as an effective treatment option for patients with UFs who desire to retain their uterus or have future pregnancies (4, 5), and it is recently suggested to improve the pregnancy rate (6). However, in some studies, a uterine rupture rate of 0.2%–3.7% is reported in women with a history of myomectomy (7). In addition, other studies have also revealed that surgical interventions, as an invasive procedure, can disrupt the physiological pelvic environment and result in pelvic adhesions, potentially decreasing the fertility of these patients (8–10). Therefore, it is urgently needed to explore an alternative non-invasive treatment modality for fertility-needing women with UFs.

As a non-invasive treatment, HIFU has been accepted by more women of childbearing age. It is indicated in recent studies that HIFU may improve the fertility of patients with UFs without increasing obstetric risks (11, 12). Fang Li et al. also held that HIFU might be an alternative fertility-sparing modality for patients with UFs (6–19). Nonetheless, relevant data are limited. Consequently, the present retrospective study was carried out aiming to compare the pregnancy outcomes of patients with UFs underwent HIFU vs. laparoscopic myomectomy (LM), and to investigate the risk factors for natural pregnancy among patients with UFs receiving HIFU or LM.

## 2 Material and methods

### 2.1 Study design and participants

In this retrospective study, totally 293 fertility-needing patients diagnosed with UFs who underwent either HIFU or LM at Nanchong Central Hospital from January 2020 to June 2023 were recruited. All patients provided informed consent prior to participation, and the study protocol was approved by the Ethics Committee of Nanchong Central Hospital. The time to pregnancy was defined as the duration from the date of surgery to the first day of the last menstrual period immediately preceding the conception cycle.

The inclusion criteria were: (1) patients with UFs in whom the diagnosis could be made using imaging modalities such as ultrasound and magnetic resonance imaging (MRI); (2) patients aged between 18 and 40 years who had fertility needs; (3) patients who had a regular sexual life and did not use contraceptive methods after surgery; and (4) patients with comprehensive clinical data and willingness to participate in follow-up assessments.

The exclusion criteria were: (1) patients with concurrent reproductive system disorders or identifiable causes of infertility, including tubal obstruction or polycystic ovarian syndrome; (2) patients with clear infertility or male factor infertility with known etiologies; (3) patients experiencing pelvic infections; (4) patients with ovarian or endocrine abnormalities; (5) patients with coexisting malignant tumors in other organs; (6) patients with allergies to contrast agents; and (7) patients unable to maintain a prone position for at least 1 h.

### 2.2 Study procedures

#### 2.2.1 HIFU group

In the HIFU group, physicians executed a uniform training program, and all patients received thorough bowel and skin preparations before the procedure. Patients in the HIFU group were treated with a sedation and analgesia regimen, and the treatment was implemented in line with the HIFU guidelines (13, 14). The JC200D1 focused ultrasound tumor treatment system (model: JC200D1; manufacturer: Chongqing Hai Fu Technology Co.) was utilized for the treatment. After surgery, each HIFU patient lied in the prone position in the observation room for 2 h. Then, they were discharged from the hospital after 1 day of observation. The women were advised to avoid conception for 1 year postoperatively.

#### 2.2.2 LM group

In the LM group, all surgical interventions were performed by the surgeons from our department who were highly trained in LM using the same technique. The procedure was carried out in line with the American College of Obstetricians and Gynecologists (ACOG) guideline (15). All patients received antibiotics for 48 h postoperatively. And the women were advised to avoid conception for 1 year postoperatively.

### 2.3 Follow-up

Following HIFU or LM treatment, all patients underwent thorough follow-up procedures. To be specific, a gynecologist conducted telephone interviews with patients on pregnancy outcomes, like pregnancy occurrence, duration, approaches, delivery modes, birth weight, and any complications experienced during pregnancy. Patients who declined to participate in the study after three attempts at follow-up assessments were classified as lost to follow-up.

### 2.4 Statistical analysis

Statistical analysis was conducted with SPSS 26.0 (SPSS, Chicago, IL, USA). Continuous nonparametric variables were presented as medians (interquartile range) and analyzed by nonparametric tests, while categorical variables were described as count (%) and examined using the chi-square test or Fisher's exact probability analysis. In addition, logistic regression analysis was employed to ascertain the factors influencing pregnancy after HIFU or LM treatment. Furthermore, the variables incorporated in the multivariate logistic regression model were selected following the Transparent Reporting of a Multivariable Prediction Model for Individual Prognosis or Diagnosis (TRIPOD) statement (16). To minimize the effects of potential confounding factors, a propensity score matching (PSM) analysis was applied, which matched patients in the two groups at a ratio of 1:1 according to age, the classification of main fibroids, the diameter of main fibroids and the number of fibroids. The two-sided *P* < 0.05 was considered significant.

## 3 Results

### 3.1 The population and pregnancy outcomes

**Figure 1:**
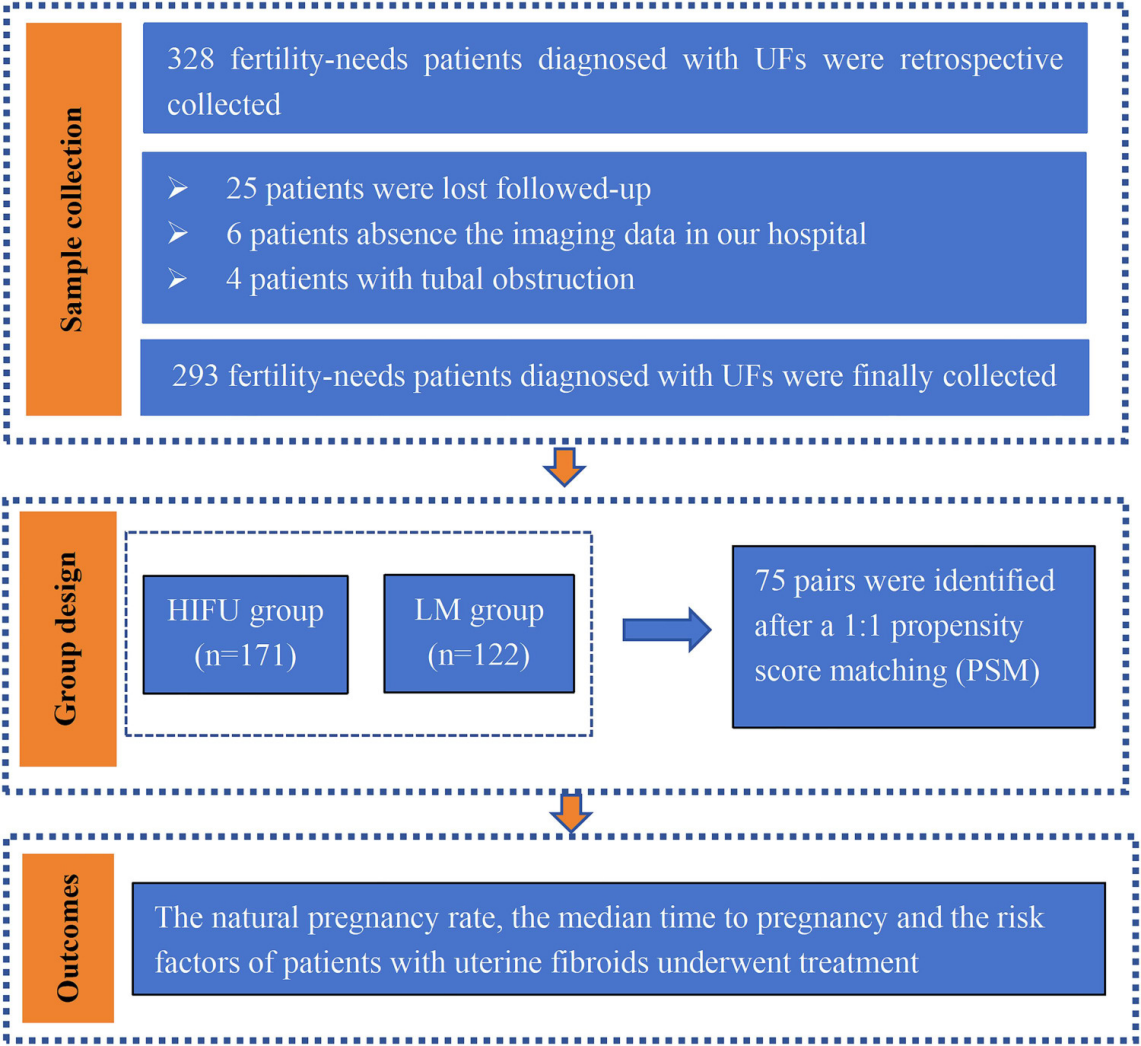
Flow chart of study participants.

During the study period, there were 328 fertility-needing patients diagnosed with UFs who received HIFU or LM treatment. Among them, 293 patients met the inclusion criteria, with 171 in the HIFU group and 122 in the LM group (Figure 1). Their follow-up data were obtained. As discovered, the median follow-up period was 30.7 (4.0–74.0) months. PSM analysis was utilized to pair the two groups at a 1:1 ratio based on factors including age, the diameter of main fibroids, the classification of main fibroids, and the number of fibroids, yielding 75 pairs (Table 1). In the unadjusted patients, the median age of the LM group was older than that of the HIFU group (33.0 years vs. 35.0 years, *P* = 0.028). After adjustment, there were no statistically significant differences in age (*p* = 0.992), body mass index (BMI) (*p* = 0.654), gravidity (*p* = 0.295), parity (*p* = 0.287), the uterine position (*p* = 0.470), the classification of main fibroids (*p* = 0.967), the location of main fibroids (*p* = 0.702), the diameter of main fibroids (*p* = 0.458) or the number of fibroids (*p* = 0.621) between the two groups (Table 1).

**Table 1:** Baseline characteristics of patients with uterine fibroids treated with HIFU or LM.

Variables	Unadjusted group			Adjusted group^b^		
	HIFU (*n* = 171)	LM (*n* = 122)	*P*-value	HIFU (*n* = 75)	LM (*n* = 75)	*P*-value
Age (years)^a^	33.0 (30.0–37.0)	35.0 (31.0–39.0)	0.028	34 (31–37)	34 (30–37)	0.992
BMI (kg/m2)^a^	21.85 (20.1–23.9)	22.37 (20.4–24.6)	0.091	22.0 (20.8–24.0)	22.3 (20.2–24.2)	0.654
BMI grade (*n*, %)			0.133			0.575
≤24	126 (75.9%)	80 (67.8%)		55 (75.3%)	52 (71.2%)	
>24	40 (24.1%)	38 (32.2%)		18 (24.7%)	21 (28.8%)	
Gravidity^a^	2.0 (1.0–3.0)	2.0 (1.0–3.0)	0.658	2.0 (1.0–3.0)	2.0 (1.0–3.0)	0.295
Parity^a^	1.0 (1.0–2.0)	1.0 (1.0–2.0)	0.159	1.0 (1.0–2.0)	1.0 (1.0–3.0)	0.287
Uterine position (*n*, %)			0.545			0.470
Anterior	96 (66.2%)	63 (70.0%)		47 (65.3%)	49 (69.0%)	
Horizon/posterior	49 (33.8%)	27 (30.0%)		24 (33.3%)	22 (31.0%)	
Classification of the main fibroids (*n*, %)^a^			0.425			0.967
Intramural	112 (73.2%)	68 (70.1%)		66 (88.0%)	65 (86.7%)	
Submucous	21 (13.7%)	19 (16.0%)		8 (10.7%)	9 (12.0%)	
Subserous	20 (13.1%)	10 (10.3%)		1 (1.3%)	1 (1.3%)	
Location of the main fibroids (*n*, %)			0.699			0.702
Anterior wall	47 (32.0%)	32 (35.6%)		22 (32.4%)	27 (38.0%)	
Posterior wall	50 (34.0%)	32 (35.6%)		26 (38.2%)	27 (38.0%)	
Other	50 (34.0%)	26 (28.9%)		20 (29.4%)	17 (24.0%)	
Diameter of the main fibroids (cm)^a^	4.90 (3.60–6.10)	5.00 (3.85–6.45)	0.602	5.1 (4.10–6.40)	4.8 (3.50–6.00)	0.458
Number of fibroids (*n*, %)			0.620			0.621
Single	55 (53.9%)	54 (57.4%)		41 (54.7%)	44 (58.7%)	
Multiple	47 (46.1%)	40 (42.6%)		34 (45.3%)	31 (41.3%)	
Total pregnancy rate (*n*, %)	43 (25.1%)	17 (13.9%)	0.017	10 (13.3%)	11 (14.7%)	0.814
Natural pregnancy rate (*n*, %)	34 (19.9%)	17 (13.9%)	0.186	10 (13.3%)	11 (14.7%)	0.814
*In Vitro* fertilization (*n*, %)	9 (20.9%)	0 (0.0%)	NA	0 (0.0%)	0 (0.0%)	NA
Median time to pregnancy (months)^a^	12.0 (9.0–15.0)	19.0 (14.5–27.0)	0.001	13.00 (9.50–14.75)	17.0 (15.0–25.0)	0.002
Abortion (*n*, %)			0.412			1.000
Spontaneous abortion	0 (0.0%)	1 (14.3%)		0 (0.0%)	1 (16.7%)	
Artificial abortion	10 (100.0%)	6 (85.7%)		4 (100.0%)	5 (83.3%)	
Pregnancies in progress (*n*, %)	0 (0.0%)	2 (11.8%)	0.022	0 (0.0%)	0 (0.0%)	
Labor			0.693			1.000
Full term delivery	27 (81.8%)	7 (87.5%)		5 (83.3%)	4 (80.0%)	
Preterm delivery	6 (18.2%)	1 (12.5%)		1 (16.7%)	1 (20.0%)	
Mode of delivery (*n*, %)			0.092			1.000
Vaginal	14 (42.4%)	1 (12.5%)		1 (16.7%)	1 (20.0%)	
Cesarean section	19 (57.6%)	7 (87.5%)		5 (83.3%)	4 (80.0%)	
Birth weight (kg)	3.0 (2.6–3.4)	3.4 (3.08–3.6)	0.194	3.0 (2.7–3.4)	3.3(3.0–3.3)	0.306
Pregnancy complications	2 (4.7%)	0(0.0%)	NA	2 (20.0%)	0(0.0%)	NA

BMI, body mass index; HIFU, high-intensity focused ultrasound; LM, laparoscopic myomectomy; NA, not applicable.

a Median (inter quartile range) values;.

b According to age, diameter of the main fibroids, classification of the main fibroids, and number of fibroids using PSM to balance the participants' characteristics between the HIFU and LM groups.

In the unadjusted patients, difference in the natural pregnancy rates between the HIFU group and the LM group was not statistically significant (HIFU vs. LM: 19.9% vs. 13.9%, *p* = 0.186), but the average time to pregnancy in the HIFU group was shorter than that in the LM group [12.0 (9.0–15.0) months vs. 19.0 (14.5–27.0) months, *p* = 0.001] (Table 1). In the adjusted patients, the nature pregnancy rates were of no significant difference between the two groups (HIFU vs. LM: 13.3% vs. 14.7%, *p* = 0.814), but the median time to pregnancy in the HIFU group was shorter than that in the LM group [13.00 (9.50–14.75) months vs. 17.0 (15.0–25.0) months, *p* = 0.002].

### 3.2 Pregnancy and delivery outcomes

In the HIFU group, 10 (13.3%, 10/75) patients became pregnant after PSM. Of them, 4 (40.0%, 4/10) patients had abortions, while 6 (60.0%, 6/10) delivered healthy babies. Moreover, 1 (16.7%, 1/6) patient had a preterm delivery, and 5 (83.3%, 5/6) had a full-term delivery. As for the choice of delivery modes, 5 (83.3%, 5/6) of them were delivered by cesarean section, and 1 (16.7%, 1/6) received vaginal delivery. Additionally, two pregnancy complications were reported, including one case of gestational diabetes and one of intrahepatic cholestasis. No uterine ruptures and placenta-related complications occurred in the HIFU group during pregnancy or delivery.

In the LM group, 11 (14.7%, 11/75) patients were pregnant after PSM. Among them, 6 (54.5%, 6/11) patients experienced miscarriage, encompassing 1 (9.1%, 1/11) undergoing spontaneous abortion, and 5 (45.5%, 5/11) suffering from artificial abortion. The rest of these patients (45.5%, 5/11) had a successful delivery, with 4 (80.0%, 4/5) cases undergoing cesarean section, while 1 (20.0%, 1/5) having vaginal delivery. One case of placenta increta was observed in this group. No others pregnancy and placenta-related complications were observed during pregnancy or delivery in the LM group.

### 3.3 Risk factors for natural pregnancy outcomes

In this study, 51 (17.4%, 51/293) cases achieved natural pregnancy after HIFU or LM treatment. There were significant differences in age (31.0 years vs. 35.0 years, *p* < 0.001), BMI (*p* = 0.037), and the classification of main fibroids (*p* = 0.011) between the two groups (Table 2). In contrast, gravidity (*p* = 0.867), parity (*p* = 0.123), the uterine position (*p* = 0.132), the location of main fibroids (*p* = 0.240), the diameter of main fibroids (*p* = 0.378), the number of fibroids (*p* = 0.897), and treatment modalities (*p* = 0.125) did not reveal any significant difference between the two groups.

**Table 2:** Baseline characteristics of patients with uterine fibroids between natural pregnancy and no pregnancy.

Variables	Natural pregnancy (*n* = 51)	No pregnancy (*n* = 233)	*P*-value
Age (years)^a^	31.0 (28.0–34.0)	35.0 (31.0–38.0)	<0.001
BMI (kg/m^2^)^a^	21.75 (20.15–22.95)	22.20 (20.7–24.56)	0.114
BMI grade (*n*, %)			0.037
≤24	42 (84.0%)	156 (69.3%)	
>24	8 (16.0%)	69 (30.7%)	
Gravidity			0.867
≤2	28 (60.9%)	128 (59.5%)	
>2	18 (39.1%)	87 (40.5%)	
Parity			0.123
≤1	35 (72.9%)	130 (61.0%)	
>1	13 (27.1%)	83 (39.0%)	
Uterine position (*n*, %)			0.132
Anterior	32 (67.8%)	124 (66.0%)	
Horizon/posterior	9 (78.0%)	64 (22.0%)	
Classification of the main fibroids (*n*, %)			0.011
Intramural	27 (60.0%)	147 (75.0%)	
Submucous	7 (15.6%)	32 (16.3%)	
Subserous	11 (24.4%)	17 (8.7%)	
Location of uterine fibroids (*n*, %)			0.240
Anterior wall	11 (23.9%)	63 (34,6%)	
Posterior wall	16 (34.8%)	65 (35.7%)	
Other	19 (41.3%)	54 (29.7%)	
Diameter of the main fibroids (cm)^a^	4.55 (3.83–6.10)	5.0 (3.77–6.20)	0.378
Number of fibroids (*n*, %)			0.897
Single	17 (54.8%)	92 (56.1%)	
Multiple	14 (45.2%)	72 (49.3%)	
Treatment modalities			0.125
HIFU	34 (66.7%)	128 (54.9%)	
LM	17 (33.3%)	105 (45.1%)	

BMI, body mass index; HIFU, high-intensity focused ultrasound; LM, laparoscopic myomectomy.

a Median (interquartile range) values.

According to our univariate logistics regression analyses, age [odds ratio (OR) = 0.873, 95% confidence interval (CI): 0.816–0.935, *p* < 0.01] and the classification of main fibroids (intramural or submucous vs. subserous: OR = 0.294, 95%CI: 0.126–0.682, *p* = 0.004) were the risk factors for natural pregnancy. Moreover, as revealed by the multivariate logistics regression analyses, age (OR = 0.899, 95%CI: 0.816–0.935, *p* < 0.001) and the classification of main fibroids (intramural or submucous vs. subserous: OR = 0.379, 95%CI: 0.158–0.910, *p* = 0.030) were the independent risk factors for natural pregnancy (Table 3). Furthermore, the multivariate logistic regression analyses demonstrated no significant differences between the treatment modalities (HIFU vs. LM: OR = 0.823, 95%CI:0.410–1.653, *p* = 0.584).

**Table 3:** Univariate and multivariate logistics regression analysis for natural pregnancy of UFs after HIFU and LM treatment.

Variables	Univariate logistics regression			Multivariate logistics regression		
	OR	95%CI	*P*-value	OR	95%CI	*P*-value
Age (years)	0.873	0.816–0.935	<0.001	0.899	0.835–0.969	0.005
BMI (kg/m^2^)	0.916	0.826–1.017	0.100			
Gravidity (>2 vs. ≤2)	0.946	0.493–1.815	0.867			
Parity (>1 vs. ≤1)	0.811	0.487–1.349	0.419			
Uterine position (horizon/posterior vs. anterior)	0.545	0.245–1.211	0.136			
The longest diameter of the uterus (cm)	0.892	0.734–1.084	0.252			
Classification of the main fibroids (intramural or submucous vs. subserous)	0.294	0.126–0.682	0.004	0.379	0.158–0.910	0.030
Location of the main fibroids (*n*, %)						
Anterior wall	ref	ref	ref			
Posterior wall	1.41	0.607–3.273	0.424			
Other	2.015	0.882–4.606	0.097			
Diameter of the main fibroids (cm)	1.038	0.973–1.107	0.260			
Number of fibroids (*n*, %)	1.052	0.486–2.276	0.897			
Treatment modalities (HIFU vs. LM)	1.641	0.868–3.102	0.128	0.823	0.410–1.653	0.584

BMI, body mass index; HIFU, high-intensity focused ultrasound; LM, laparoscopic myomectomy, ref, reference.

## 4 Discussion

In the retrospective study, no significant difference were observed in natural pregnancy rates between women with UFs undergoing HIFU ablation vs. LM treatment, both before and after PSM analysis. However, the HIFU group exhibited a significantly shorter median time to pregnancy than the LM group after adjustment. These findings suggest that HIFU, as a non-invasive treatment procedure, may represent an alternative option for fertility-needing women with UFs. Nevertheless, large-scale prospective studies are warranted to confirm these results.

Currently, several retrospective studies have reported comparable pregnancy rates between women receiving HIFU and those undergoing LM (17, 18). As found in the meta-analysis carried out by Li et al., there were similar pregnancy rates between ultrasound-guided high intensity focused ultrasound (USgHIFU) and myomectomy, with pregnancy rates of 35% and 43%, respectively (19). A comparable trend was also observed in our study. Notably, the natural pregnancy rates in our study were relatively lower than the previously reported ones, with rates of 19.9% in the HIFU group and 13.9% in the LM group (19). This may be interpreted from the following aspects. Firstly, our study included women younger than 40 years with fertility needs, but this population may still include individuals with age-related declines in fertility, particularly those in their late reproductive years (20, 21). Secondly, a relatively high proportion of multipara were included in our study (parity ≥ 1). These women expressed a desire to preserve fertility but did not actively attempt to conceive during the follow-up period. Finally, previous studies have indicated that fibroid size and location are the critical determinants of fertility outcomes and a substantial proportion of women in our study fell into this category (22).

In addition, our study demonstrated that the HIFU group had a significantly shorter median time to pregnancy than the LM group [13.00 (9.50–14.75) months vs. 17.0 (15.0–25.0) months, *p* = 0.002], consistent with previous findings (17, 18). The possible mechanisms are lacking. First of all, as a non-invasive treatment modality, HIFU treatment does not penetrate the myometrium or the uterine serosa. This can minimize the risk of uterine scarring and the formation of pelvic or abdominal adhesions (23). Besides, HIFU offers a faster postoperative recovery than LM, allowing patients to initiate attempts at conception earlier (24).

Age has been identified as the main risk factor for pregnancy, which may affect the natural pregnancy in patients with UFs undergoing HIFU or LM treatment (25). Consistently, our study supported that age was an independent risk factor for the natural pregnancy of patients with UFs who underwent HIFU or LM. The main reason is that with increasing age, particularly after ≥35 years, the ovarian reserve function declines substantially, leading to the reduced implantation rate of fertilized eggs (20). Furthermore, the location of UFs is the other main factor influencing natural pregnancy among patients with UFs receiving HIFU or LM. Specifically, compared with subserosal fibroids, intramural and submucosal fibroids have a greater impact on natural fertility after surgery (22). However, the possible mechanism remains to be further explored. First, compared with UFs of other locations, subserosal fibroids may have less impact on uterine cavity anatomy and endometrial receptivity, thus achieving optimal restoration of uterine cavity anatomy and endometrial receptivity after HIFU or LM (26). Second, the fastest recovery of utero-tubal peristalsis and sperm transport can be achieved due to limited involvement of the myometrial junctional zone (27).

Nonetheless, there are still certain limitations to be noted in this study. Firstly, the sample size of this study was limited, and the stratified analyses should be further refined. Prospective multicenter studies are warranted in the future. Secondly, we were unable to obtain data on the sexual activity frequency of patients, which may have introduced some selection bias considering the retrospective nature of this study. In addition, Because of the limited sample size, the FIGO classification system was not applied. Nevertheless, the conventional classification into submucosal, intramural, and subserosal fibroids may partially reflect the influence of fibroid location on reproductive outcomes. Finally, endometrial blood perfusion and endometrial receptivity were not assessed in this study, and further studies should be conducted to investigate the potential mechanism after HIFU treatment.

In conclusion, HIFU demonstrated comparable postoperative pregnancy rates to LM and was associated with a shorter time to conception, suggesting that HIFU may be a potential fertility-sparing treatment for women with UFs. Additionally, age and the location of dominant fibroids were identified as the independent factors influencing postoperative pregnancy rates in patients undergoing treatment.

## Data Availability

The raw data supporting the conclusions of this article will be made available by the authors, without undue reservation.
